# Lactate stimulates CA IX expression in normoxic cancer cells

**DOI:** 10.18632/oncotarget.20836

**Published:** 2017-09-12

**Authors:** Elena Panisova, Martin Kery, Olga Sedlakova, Lucie Brisson, Michaela Debreova, Martina Sboarina, Pierre Sonveaux, Silvia Pastorekova, Eliska Svastova

**Affiliations:** ^1^ Institute of Virology, Biomedical Research Center, Slovak Academy of Sciences, Bratislava, Slovakia; ^2^ Unit of Pharmacology and Therapeutics, Institut de Recherche Expérimentale et Clinique (IREC), Université Catholique de Louvain (UCL), Brussels, Belgium; ^3^ Inserm UMR1069, Nutrition, Croissance et Cancer, Université François-Rabelais, Tours, France

**Keywords:** tumor metabolism, carbonic anhydrase IX, lactate, HIF-1, MCT1

## Abstract

Besides hypoxia, other factors and molecules such as lactate, succinate, and reactive oxygen species activate transcription factor hypoxia-inducible factor-1 (HIF-1) even in normoxia. One of the main target gene products of HIF-1 is carbonic anhydrase IX (CA IX). CA IX is overexpressed in many tumors and serves as prognostic factor for hypoxic, aggressive and malignant cancers. CA IX is also induced in normoxia in high cell density. In this study, we observed that lactate induces CA IX expression in normoxic cancer cells *in vitro* and *in vivo*. We further evidenced that participation of both HIF-1 and specificity protein 1 (SP1) transcription factors is crucial for lactate-driven normoxic induction of the *CA9* gene. By inducing CA IX, lactate can facilitate the maintenance of cancer cell aggressive behavior in normoxia.

## INTRODUCTION

Cancer cells have to change metabolic pathways to fulfill their high bioenergetic and biosynthetic needs in conditions of poor oxygen and nutrient availability found in the tumor microenvironment. This is possible through the high metabolic plasticity of cancer cells which use glycolysis in conditions of reduced oxygen supply but also in the presence of oxygen (Warburg effect) [1]. Despite of extensive use of aerobic glycolysis by cancer cells, oxidative metabolic activities are sustained and provide them with a broad spectrum of advantages [2]. The metabolic byproducts in turn influence metabolic and signaling pathways of proximal, as well as distal tumor areas [3]. Lactate, the final product of glycolysis of hypoxic cells, is exported and diffused to an oxygenated tumor niche, where it is used for oxidative metabolism and saves glucose for areas with insufficient oxygen supply [4]. *In vivo* experiments in rat mammary carcinomas proved that lactate is preferentially taken-up in well-perfused tumor regions without hypoxia [5] supporting the model of metabolic symbiosis [4]. Further reports have shown that the cooperation between glycolytic and oxidative cancer cells facilitated by lactate goes far beyond energetic advantage. The conversion of lactate to pyruvate via LDHB provides NADH and H^+^ that promote VATPase dependent lysosomal acidification and high autophagic flux in oxidative cancer cells [6]. The use of antiangiogenic therapy also resulted in a metabolic symbiosis between arising normoxic and hypoxic clusters of cancer cells in tumors. Normoxic cells surrounding vessels express MCT1, import and metabolize lactate while cells in hypoxic clusters express GLUT1, MCT4 and import and metabolize glucose [7]. Lactate is also able to stimulate the production of reactive oxygen species (ROS), increase DNA binding of ROS-responsive transcription factors [8] and stabilize α subunit of transcription factor hypoxia-inducible factor-1 (HIF-1α) [9–13].

HIF-1 is an important regulator that considerably changes the transcriptional profile of hypoxic cancer cells in order to promote their survival in stressful conditions of oxygen deprivation [14]. HIF-1 is a heterodimer that consists of a constitutively expressed β subunit and an O_2_-regulated α subunit [15]. In normoxia, hydroxylation of two proline residues in the HIF-1α O_2_-dependent degradation domain (ODDD) by O_2_-dependent prolyl hydroxylases (PHDs) leads to von Hippel Lindau (VHL)-dependent polyubiquitylation of HIF-1α and its degradation by the proteasome. In hypoxia, inactivation of PHDs results in HIF-1α stabilization [16]. Even though hypoxia represents the primary stimulus driving HIF-1α accumulation, HIF-1α was also observed in oxygenated tumor areas and metastatic nodules [17], suggesting that other mechanisms (in addition to O_2_ deficiency) regulate HIF-1α stabilization and HIF-1 activation.

The HIF-1 pathway represents the integrator and mediator of signals leading to activation of the *CA9* gene that encodes carbonic anhydrase IX (CA IX) [18, 19]. CA IX is a transmembrane protein that preferentially catalyzes the reaction CO_2_ + H_2_O –> HCO_3_^-^ + H^+^[20, 21]. By cooperating with anion exchanger 2 (AE2) and Na^+^/bicarbonate cotransporter 1 (NBCe1) [22], CA IX serves as a pH regulatory component that provides acid-base balance. It is an important factor of tumor progression and participates in cell-cell de-adhesion, stimulation of migration and invasion, and formation of focal contacts [22–24]. The contribution of CA IX to the acquisition of more advanced tumor phenotypes is supported by its association with higher tumor grades and relapse rates, relation to poorly differentiated and greatly invasive tumors, and worse overall and progression-free survival in patients [25].

*CA9* gene transcription can be highly induced by hypoxia in a HIF-dependent manner, which is supported by the overlap of pimonidazole and CA IX staining in hypoxic regions of tumors [18]. However, CA IX distribution in tumors does not always correlate with other hypoxic markers, such as HIF-1α, glucose transporter 1 (GLUT1) and vascular endothelial growth factor (VEGF) [26]. One of the alternative mechanisms of CA IX regulation is driven by normoxic high cell density. Indeed, CA IX is not expressed in sparse HeLa cells, but is rapidly induced in dense cell cultures, with the contribution of pericellular hypoxia (1 – 5%) formed above the dense cell layer [10, 27, 28]. *CA9* gene expression in high cell density requires minimal levels of active HIF-1α and SP1 transcription factors. Moreover, SP1 represents a central component and integrator of pathways leading to *CA9* induction in dense cultures [28].

In this study, we focused on the ability of lactate to promote normoxic expression of CA IX protein, generally associated with hypoxic tumors. We report that lactate stimulates CA IX expression through HIF-1α stabilization independently of hypoxia. Further, we identify *in vivo* codistribution of CA IX with MCT1, lactate monocarboxylate transporter 1, indispensable for paracrine lactate activity. Thus, CA IX can be one of the crucial effectors of lactate which represents a key metabolic microenvironmental factor enhancing cancer cell aggressiveness.

## RESULTS

### Lactate increases HIF-1α protein levels in normoxia

**Figure 1:**
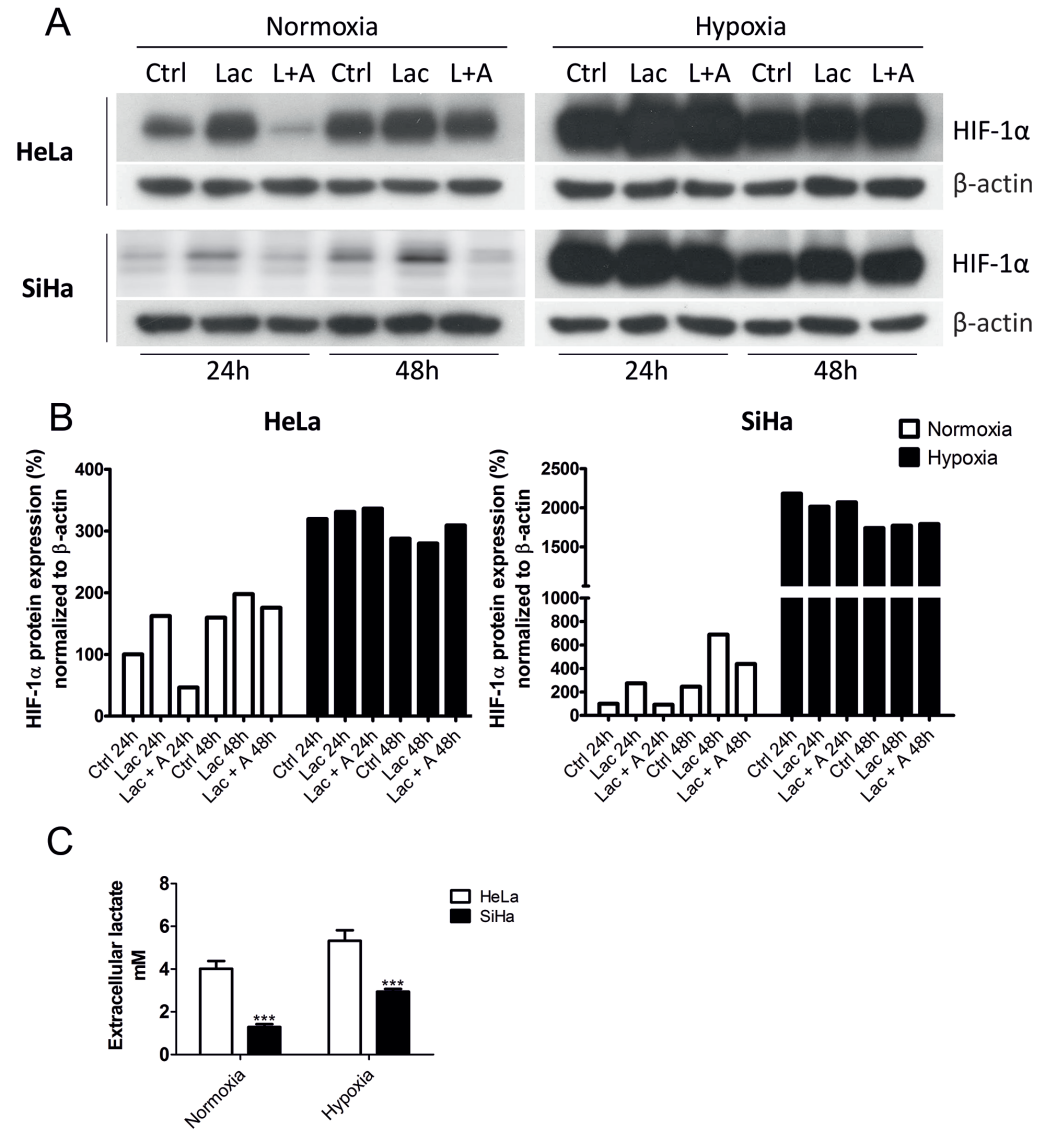
Lactate stabilizes HIF-1α protein levels in normoxic HeLa and SiHa tumor cells HIF-1α protein was detected by Western blot after 24 h and 48 h normoxic and hypoxic (2%) incubation in the control media (Ctrl) supplemented with 10 mM sodium lactate (Lac) or 10 mM sodium lactate combined with 100 μM sodium ascorbate (L/Lac + A) in HeLa and SiHa cells **(A)**. HIF-1α protein was quantified after normalization to β-actin in HeLa and SiHa cells. 24 h Ctrl represents 100% **(B)**. Extracellular lactate (mM) of HeLa and SiHa cells (n = 3) was measured in control media after 24 h incubation in normoxia and hypoxia. Averaged data from 3 independent experiments are shown **(C)**. All data represent means ± SEM, ***p < 0.005, compared to nontreated control (Ctrl) cells after 24 h by two-tailed Student´s *t* test.

It has been previously shown that lactate can stabilize HIF-1α expression in a broad spectrum of cell lines [11–13]. The exact mechanism is currently unknown, but one possibility lies within the regulation of the cell redox state, because ascorbate (a PHD cofactor important for the reduction of Fe^3+^ to Fe^2+^ to maintain PHD activity) reverts lactate-induced HIF-1α stabilization [11]. First, we decided to verify that redox changes participate in lactate-induced HIF-1α stabilization in our experimental settings. HeLa and SiHa cells were treated for 24 h and 48 h with 10 mM of sodium *L*-lactate with or without 100 μM of sodium *L*-ascorbate dissolved in nutrient-reduced cell culture medium (1 g/L of glucose, 1% FCS, 10 mM NaHCO_3_, no pyruvate) in order to reproduce microenvironmental conditions of tumors. The concentration of lactate in tumors ranges from 4 to 40 μmol/g, with an average of about 15 μmol/g [29, 30]. We decided to use a 10 mM concentration of lactate. For ascorbate, a 100 μM concentration was chosen due to previously confirmed effects on lactate-stabilized HIF-1α [11]. We used sodium salts to avoid acidification, which on its own has a striking impact on cell signaling pathways [31, 32]. We also avoided adding pyruvate to our media because the conversion of lactate to pyruvate is inevitable for its effect on HIF-1α stabilization [11]. We observed that lactate increases HIF-1α protein levels in both HeLa and SiHa cells after 24 h and 48 h in normoxia, whereas hypoxic induction of HIF-1α was lactate-independent (Figure 1A, 1B). Comparison of HeLa and SiHa cells revealed much higher basal (normoxic) HIF-1α protein levels in HeLa (Figure 1A, 1B). This is in agreement with previous observations that cells with high rates of aerobic glycolysis, including HeLa cells, express high basal HIF-1α levels [10, 11]. In our experimental conditions, HeLa released 3-times more lactate to the culture medium than SiHa cells (Figure 1C). Providing exogenous lactate during normoxia increased HIF-1α level 162-times in HeLa and 273-times in SiHa cells compared with its basal HIF-1α levels (Figure 1A, 1B). Both cell lines demonstrated similarly huge increase in HIF-1α protein in hypoxia (Figure 1A, 1B). Addition of ascorbate reverted basal but not hypoxia-induced HIF-1α accumulation and confirmed the oxygen-independent mechanism of HIF-1α regulation by lactate (Figure 1A, 1B).

### Lactate increases mRNA levels of HIF-1-target genes *CA9*, *SLC16A3*/*MCT4*, *LDHA* and *VEGFA*

**Figure 2:**
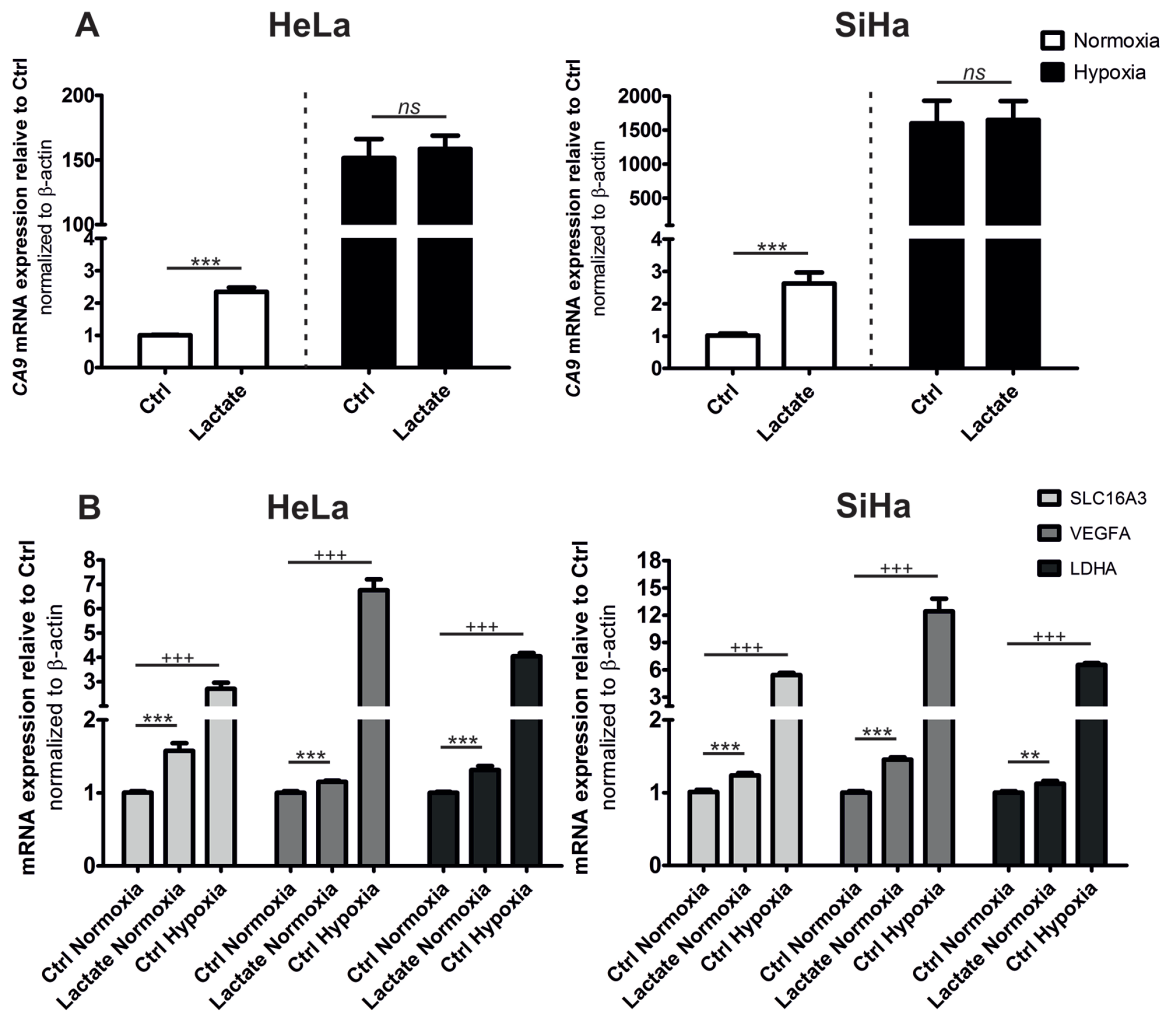
Lactate increases CA9 mRNA to the highest rate compared to other HIF-1 target genes qRT-PCR analysis of HIF-1 target genes CA9 **(A)** and SLC16A3/MCT4, VEGFA, LDHA **(B)** after 24 h normoxic or hypoxic incubation with or without 10 mM sodium lactate compared to control conditions (Ctrl) in HeLa and SiHa tumor cells. Data were calculated in relation to Ctrl normoxia for every gene separately. Averaged data from 3 independent experiments are shown (n = 3). All data represent means ± SEM, **p < 0.01, ***p < 0.005, ns, not significant by two-tailed Student´s t test.

HIF-1regulates the transcription of a broad spectrum of genes involved in cellular processes including cancer cell progression, angiogenesis and glycolysis, with subsequent lactate efflux [33–35]. Because lactate stabilized HIF-1α in our experimental conditions, we decided to study the effects of lactate on the expression of several HIF-1-target genes in HeLa and SiHa cells using RT-qPCR. We also compared the transcription of selected genes after cell stimulation with lactate or hypoxia. We found that lactate increased *CA9* transcription more than 2-fold in HeLa (2.35-fold induction) and SiHa (2.63-fold induction) cells in normoxic conditions (Figure 2A). Dramatic upregulation of *CA9* mRNA level was expectantly induced by hypoxia-stabilized HIF-1α (Figure 2A). However, we observed no response to lactate on *CA9* mRNA in hypoxia.

The influence of lactate on the expression of HIF-1α-target genes *SLC16A3*, *LDHA*, and *VEGFA* was much smaller than on *CA9*. In HeLa cells, 24 h of lactate stimulation increased mRNA levels of *SLC16A3* and *LDHA* (1.58- and 1.31-fold increases) (Figure 2B). The most remarkable induction in SiHa cells, except *CA9*, was observed for *VEGFA* mRNA (1.46-fold induction) (Figure 2B).

In our models, the hypoxic stimulation of *SLC16A3*, *LDHA* and *VEGFA* transcription was also much less pronounced in comparison to hypoxic induction of *CA9* (Figure 2).

### Lactate increases CA IX protein levels in normoxia

**Figure 3:**
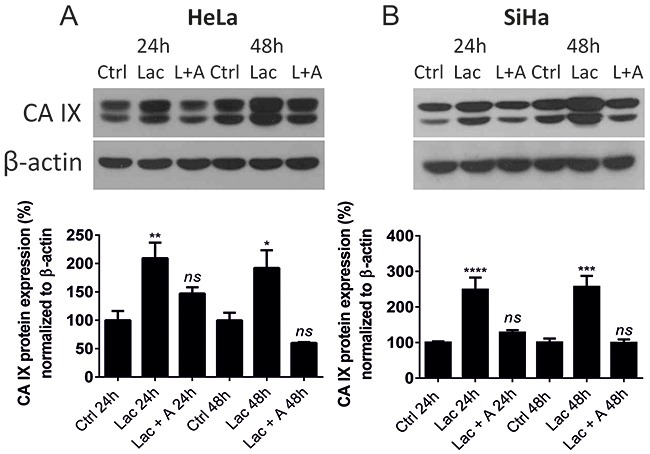
Lactate elevates CA IX protein levels in HeLa and SiHa tumor cells CA IX protein was detected by Western blot and quantified after normalization to β-actin after 24 h and 48 h normoxic incubation in the control media (Ctrl) supplemented with 10 mM sodium lactate (Lac) or 10 mM sodium lactate combined with 100 μM sodium ascorbate (L/Lac + A) in normoxic HeLa **(A)** and SiHa cells **(B)**. 24 h Ctrl and 48 h Ctrl represent 100%. Averaged data from 3 independent experiments are shown. All data represent means ± SEM, *p < 0.05, **p < 0.01, ****p < 0.0001, *ns*, not significant compared to nontreated control (Ctrl) cells after 24 h or 48 h by one-way ANOVA test.

Having shown that *CA9* mRNA expression is induced by lactate, we further verified that the effect was also detected at the protein level. We treated cells with 10 mM *L*-lactate with or without 100 μM ascorbate for 24 h and 48 h, and determined CA IX protein levels by immunoblotting. Lactate increased CA IX protein expression in HeLa (Figure 3A) and SiHa (Figure 3B) cells after 24 h and 48 h, with a greater effect on SiHa cells. Ascorbate blocked lactate-stimulated CA IX induction, which indicated that the major mechanism of lactate on CA IX was through redox-dependent HIF-1α stabilization.

### Lactate primarily controls *CA9* gene expression through HIF-1 in cooperation with SP1 and AP1

**Figure 4:**
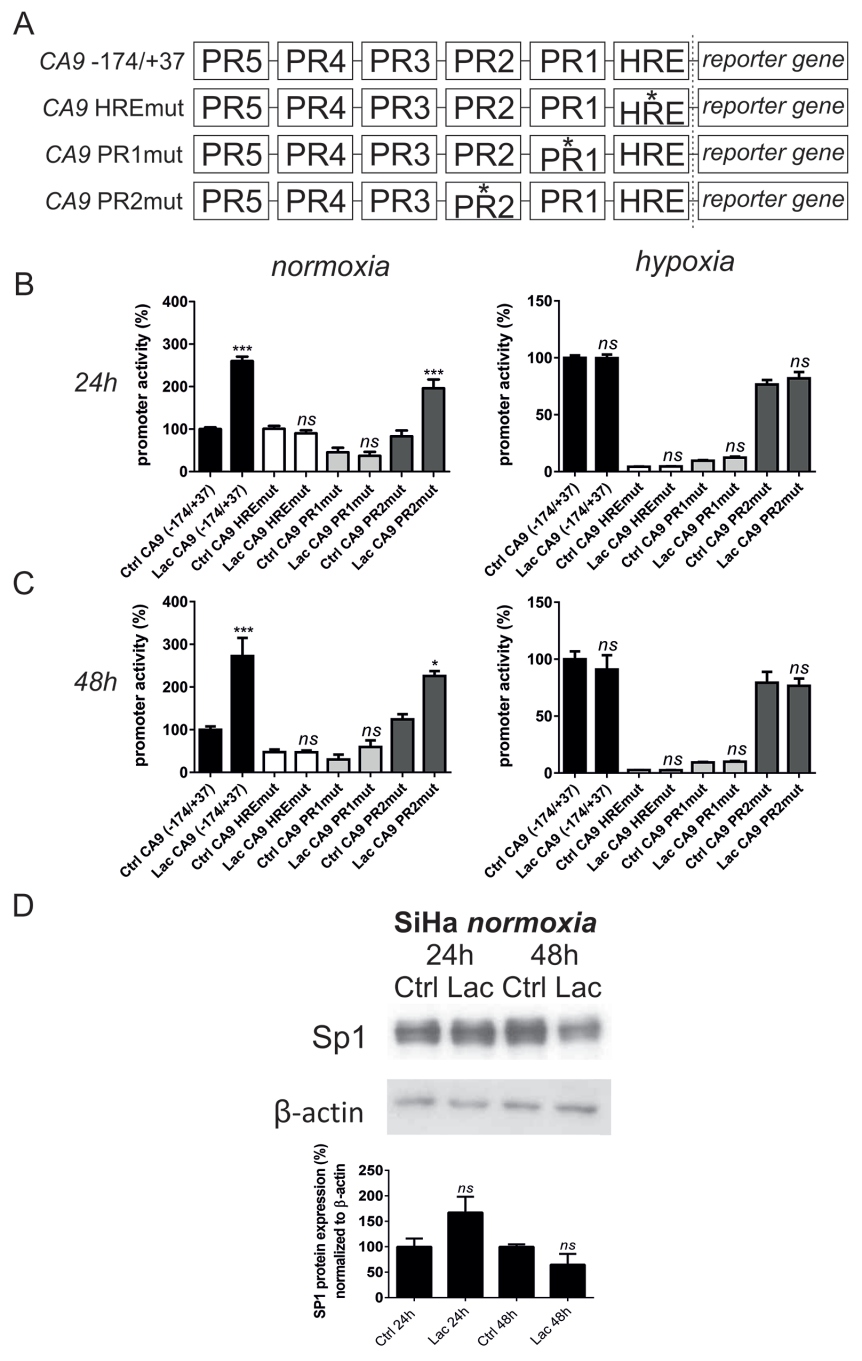
Lactate stimulates *CA9* expression by coordinating both HIF-1 and SP1 transcription factors in SiHa cells The transcriptional activity of *CA9* promoter (-174/+37) was determined using a dual luciferase reporter assay in SiHa cells. **(A)** SiHa cells were transfected with 4 different promoter constructs: wild type *CA9* promoter (-174/+37); promoter (-174/+37) with mutation in HRE element within HIF-1 binding site (*CA9* HREmut); promoter (-174/+37) with mutation in PR1 element within SP1 binding site (*CA9* PR1mut); and promoter (-174/+37) with mutation in PR2 element within AP1 binding site (*CA9* PR2mut). Transfected SiHa cells were treated with 10 mM sodium lactate for 24 h **(B)** and 48 h **(C)** in normoxia and 2% hypoxia with subsequent measurement of reporter gene luciferase activity. Averaged data from 3 independent experiments are shown (n = 4). **(D)** SP1 protein was detected by Western blot and quantified after normalization to β-actin after 24 h and 48 h normoxic incubation in the control media (Ctrl) or with addition of 10 mM sodium lactate (Lac) in SiHa cells. 24 h Ctrl and 48 h Ctrl represent 100%. Averaged data from 3 independent experiments are shown. All data represent means ± SEM, *p < 0.05, ***p < 0.005, *ns*, not significant compared to nontreated control (Ctrl) cells of each promoter construct separately after 24 h or 48 h by one-way ANOVA.

The *CA9* gene contains six *cis*-regulatory regions (Figure 4A), including a hypoxia-response element (HRE) and five protected regions PR1 – PR5 [18, 36]. HRE is recognized by HIF-1 [18]. This region is the most critical, but full transcriptional activation of the *CA9* gene requires the cooperation of HIF-1 with the SP1 transcription factor that binds to the PR1 region [37]. Active SP1 was shown to be a central component and integrator of pathways involved in high cell-density regulation of the *CA9* gene [28]. PR2 is an AP1-binding site and is an important for enhancement of *CA9* transcriptional level [38, 39]. We used wild-type (wt) and 3 different mutants of the *CA9* promoter (-174/+37), preventing the binding of HIF-1, SP1 or AP1 (Figure 4A). We determined their transcriptional activity by a dual luciferase assay upon lactate treatment for 24 h and 48 h in normoxia and 2% hypoxia. We decided to use SiHa cells only due to the more pronounced effect of lactate on CA IX protein levels in this cell line.

Elimination of the SP1-binding site of the PR1 in the *CA9* promoter led to a more severe decrease in the promoter activity in normoxia (54.5% lower activity of PR1mut after 24 h, and 70% lower activity of PR1mut after 48 h compared to wt control promoter) in comparison to mutation of the HIF-1-binding site HRE (0.73% difference between HREmut and wt control promoter after 24 h; 52.7% lower activity of HREmut compared to the wt control promoter after 48 h). Under hypoxia, mutation of HRE resulted in more dramatic effects compared to the PR1 mutation (2.25 x higher promoter activity of PR1mut compared to HREmut after 24 h and 3.73 x after 48 h). These results reflect differences in the mechanisms driving CA IX expression under hypoxia and mild hypoxia derived from high cell-density. Thus, CA IX induction relies more on SP1 activity in confluent cells (mild hypoxia), whereas HIF-1 predominates under strong hypoxia.

Lactate increased the activity of the wt *CA9* promoter after 24 h (Figure 4B) from 100% to 260.07% (p < 0.005) and after 48 h (Figure 4C) to 272.6% (p < 0.005) in normoxia only, but it exhibited no effect on hypoxic *CA9* promoter induction. This indicates that extracellular lactate is relevant in normoxic *CA9* stimulation, whereas in hypoxia, lack of oxygen predominates. We further showed that the mutation of HRE, as well as of PR1, prevented lactate from stimulating the *CA9* promoter after 24 h and 48 h. Having shown that lactate increases HIF-1α protein levels (Figure 1), we next examined whether lactate stimulates SP1 protein levels, thereby participating in *CA9* induction not only by affecting HIF-1 but also SP1. Figure 4D shows that exogenous lactate did not significantly increase SP1 protein levels after 24 h, and even caused a drop in SP1 after 48 h of incubation. These data point to the importance of HIF-1-SP1 coordination in *CA9* induction, rather than at the regulation of SP1 protein levels by lactate. Mutation of the AP1 binding site in the PR2 (Figure 4B and 4C) led to a minor decrease of the lactate effect on *CA9*, which might reflect its role as a *CA9* gene enhancer.

### Lactate increases CA IX protein levels in SiHa tumor xenografts

**Figure 5:**
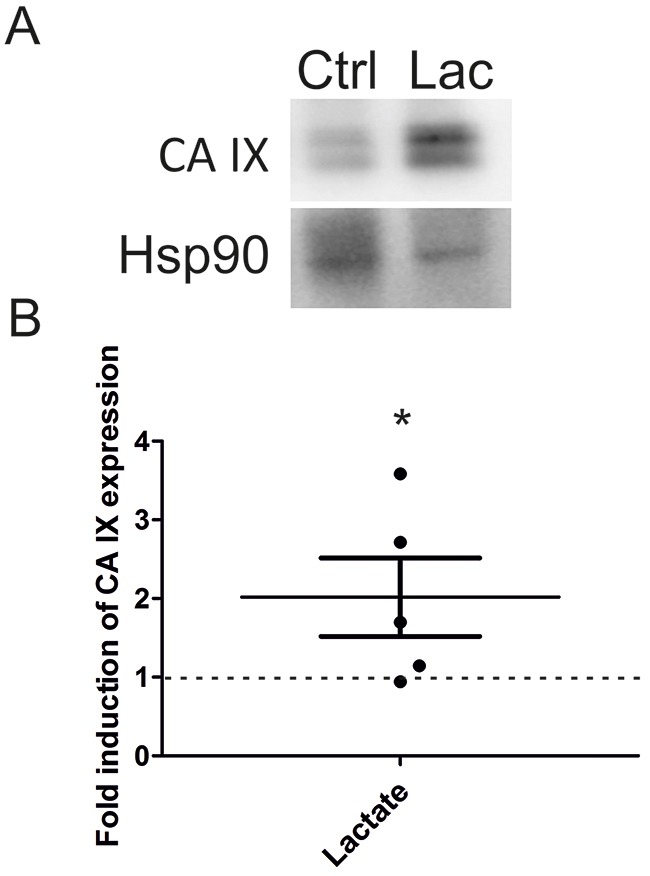
Lactate increases CA IX level in SiHa xenografts **(A)** Representative immunoblot of CA IX and Hsp90 as an endogenous control in tumors collected 12 days after being established with Matrigel plugs in mice with SiHa cells containing 30 mM lactate (Lac) or containing an equal volume of saline (Ctrl). **(B)** Graph represents fold induction of CA IX expression detected by Western blot and quantified by ImageJ software after normalization to Hsp90 in 5 individual tumors. Dashed line (1) constitutes the value for control tumors (n = 5). All data represent means ± SEM, *p < 0.05 compared to control by Student's *t* test.

In order to validate the above *in vitro* observations, we decided to examine the effect of lactate on CA IX protein expression *in vivo*. For this purpose, we used SiHa xenografts prepared and described previously [40]. Xenografts were grown from 10^6^ subcutaneously-injected SiHa cells in growth factor-reduced Matrigel containing 30 mM of sodium *L*-lactate (right flank) or an equal volume of saline (left flank). Dissected Matrigel plugs were snap-frozen and protein expression was analyzed by immunoblotting. Figure 5A depicts a representative immunoblot of CA IX and Hsp90 as an endogenous control. Fold induction of CA IX in individual lactate-containing Matrigel plugs compared to control plugs (value 1 – dashed line) is depicted in Figure 5B. The average induction of CA IX by lactate was 2.02 compared to control value 1 (p < 0.05).

### MCT1 and CA IX are partly codistributed in tumors *in vivo*

**Figure 6:**
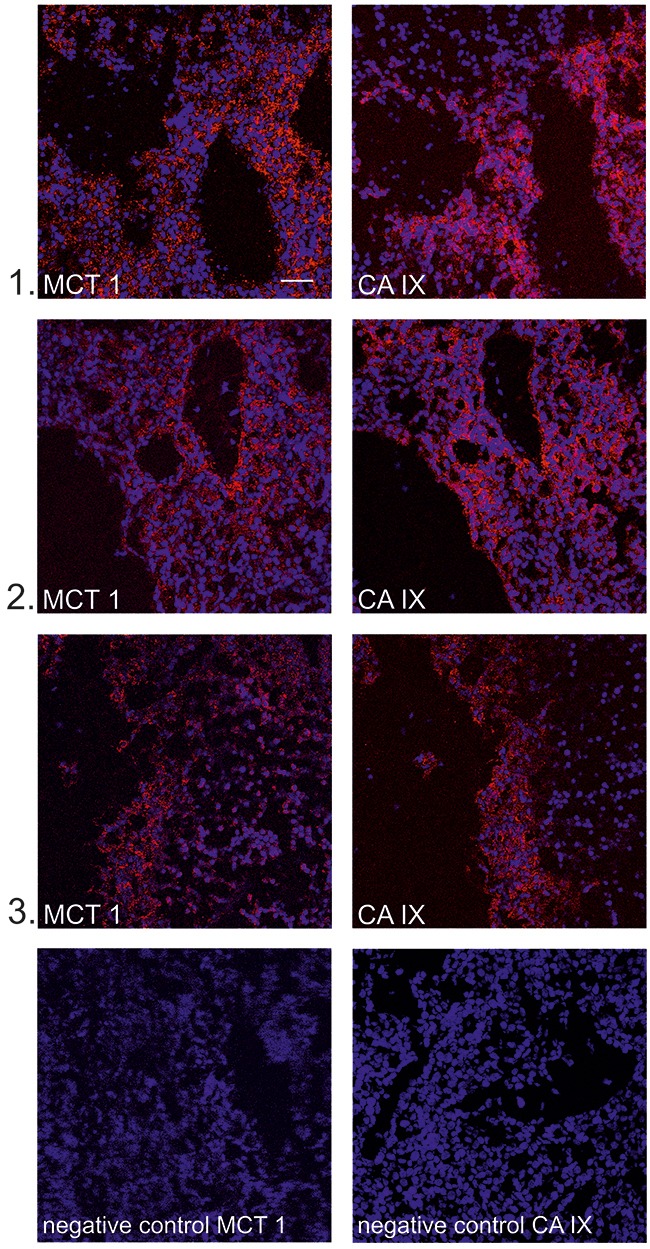
MCT1 and CA IX partially colocalize in cryosections of SiHa xenografts Representative confocal images **(1-3)** show fluorescent staining of MCT 1 (red, left panel) and CA IX (red, right panel) and nuclei (blue) in cryosections from SiHa xenografts. MCT1 and CA IX staining was performed on consecutive sections and corresponding areas were identified by microscopy. Secondary antibodies with omission of the primary antibodies against MCT1 and CA IX were used as negative controls. Scale bar 50 μm.

MCT1 is a passive lactate-H^+^ symporter enabling the uptake of lactate by oxidative cancer cells [4]. Because it facilitates lactate uptake by cancer cells, MCT1 represents a key component of lactate-induced HIF-1 activation, and its inhibition blocks lactate-mediated effects [9, 12, 32, 41]. To get a deeper insight into the relationship between MCT1 and CA IX in the tumor microenvironment, we analyzed the relative localization of these two proteins in lactate-treated SiHa xenografts. We stained two consecutive SiHa tumor sections. Representative images revealed that MCT1 and CA IX were present in the same tumor areas (Figure 6). Codistribution of CA IX and MCT1 in the same regions of lactate-treated xenografts supports the function of lactate as a microenvironmental factor inducing expression of HIF-1α target genes in normoxic areas of tumors.

**Figure 7:**
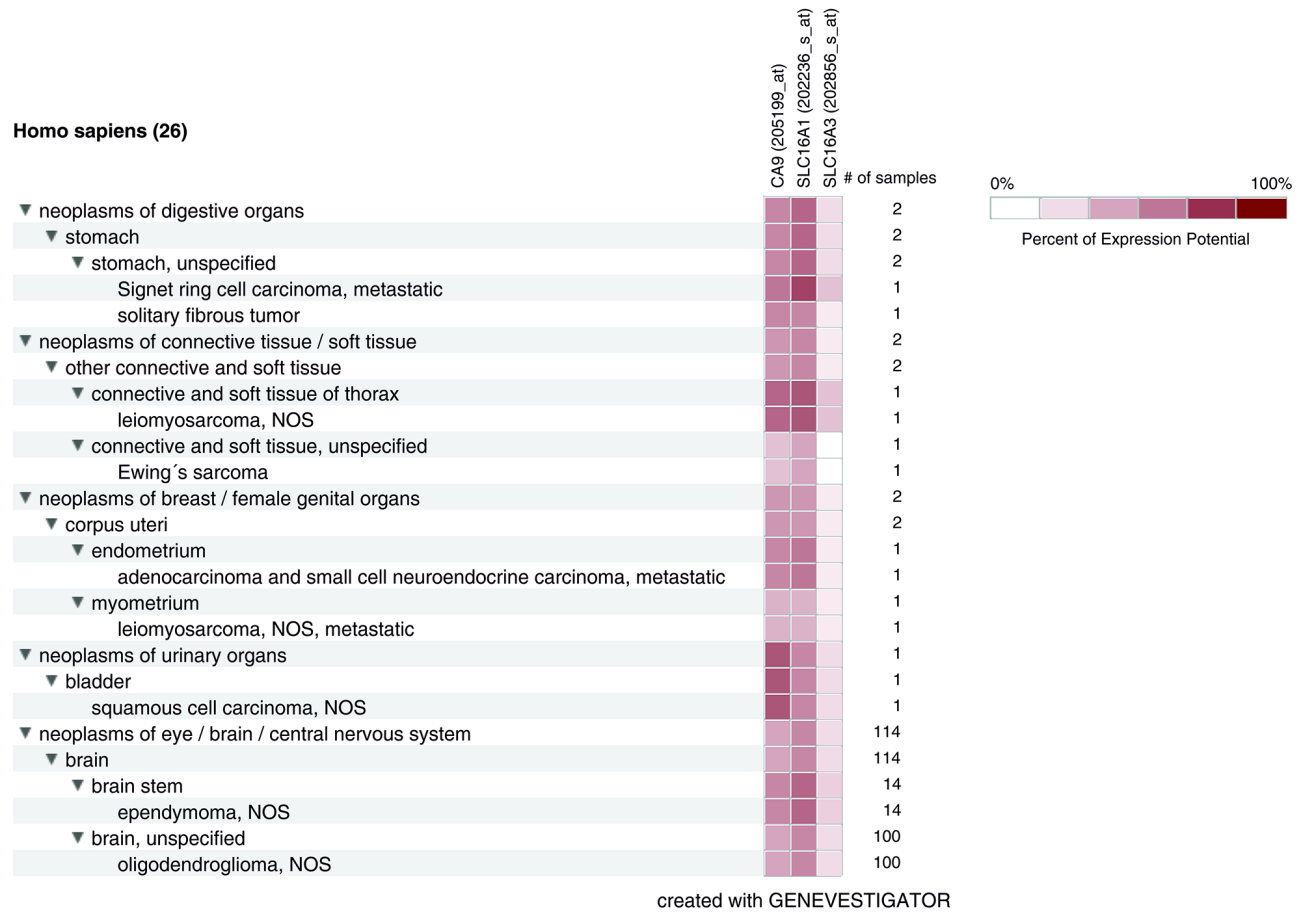
MCT1 and CA IX are coexpressed in neoplasms with low MCT4 expression Heatmap Tree of expression patterns of *CA9* (encoding CA IX), *SLC16A1* (encoding MCT1), and *SLC16A3* (encoding MCT4) genes. Different types/origins of neoplasms are shown and listed on the left. Number of samples are listed on the right.

We further employed Genevestigator analysis to get an overview of the expression levels of cancer-specific genes in various neoplasms [42]. Publicly available microarray experiments were analyzed for high *CA9* and *SLC16A1/MCT1* expression and concurrently low *SLC16A3/MCT4* expression (Figure 7). Positive results involve several neoplasms including Stomach Signet ring cell carcinomas; connective/soft tissue neoplasms such as leiomyosarcomas and Ewings's sarcomas; uterine neoplasms such as adenocarcinomas and endometrial small cell neuroendocrine carcinomas and myometrialleiomyosarcomas; bladder squamous cell carcinomas; and brain neoplasms such as oligodendromas and pediatric ependymomas. All of these cases were malignant tumors, except for one benign solitary fibrous tumor of the stomach. Signet ring cell carcinomas, myometrialleiomyosarcomas, as well as endometrial adenocarcinomas and small cell neuroendocrine carcinomas were metastatic. The majority of the cases for which correlation was found were within the group of brain tumors, particularly oligodendromas (n=100) and ependymomas (n=14). Eighty seven oligodendroma cases were graded: 29 according to histopathological classification (18x grade II; 11x grade III) and 58 according to World Health Organization (WHO) grading (44x grade 2; 14x grade 3). Ependymomas were divided based on WHO grading into grade 2 (10 cases) and grade 3 (4 cases).

## DISCUSSION

In this study, we confirm that lactate stabilizes HIF-1α in HeLa and SiHa cancer cell lines, which is in good agreement with previous findings [9]. In the presence of oxygen, PHD dioxygenases initiate the degradation pathway of HIF-1α. In addition to oxygen, they require reduced ferrous iron (Fe^2+^) and 2-oxoglutarate (2-OG) for their activity [43]. Moreover, ascorbate is needed as a cofactor to regenerate Fe^2+^ from Fe^3+^ produced during HIF-1α hydroxylation [11, 16]. Ascorbate has been previously shown to reverse basal but not hypoxia-induced HIF-1α accumulation [11, 13, 44]. We confirm those findings here by evidencing that ascorbate blocks lactate-induced HIF-1α stabilization in both HeLa and SiHa cells but hypoxia-induced HIF-1α stabilization is resistant to the ascorbate-dependent degradation activity of PHDs. Others have shown that ascorbate supplementation in the range of 25 - 400 μM has no effect on hypoxic induction of HIF-1α in vascular smooth muscle cells [45]. Moreover, Knowles et al. revised the mechanism of ascorbate action on HIF PHD activity. In conditions of PHD blockade by an oxoglutarate analogue, HIF-1α protein accumulates and ascorbate has no effect on HIF-1α levels. Interestingly, the impact of ascorbate on HIF-1α accumulation can be partially dependent on the degree of hypoxia, with strongest effects observed at high O_2_ tensions [44]. The exact mechanism by which lactate stabilizes HIF-1α is still unknown, but it has been reported that lactate (or more precisely pyruvate produced from lactate oxidation) competes with 2-OG for PHDs [11]. Indeed, pharmacological inhibition of LDH-B (the enzyme converting lactate to pyruvate) or LDH-B silencing prevented lactate-induced HIF-1α accumulation in normoxic conditions [12]. Pyruvate could therefore directly interact with the active site of the enzyme. Another possibility lies within ROS that oxidize Fe^2+^ to Fe^3+^ and inactivate PHDs, thereby stabilizing HIF-1α [46]. Lactate stimulates ROS production (presumably by promoting mitochondrial respiration) and could therefore inactivate PHDs and stabilize HIF-1α via this mechanism [8].

HIF-1 is a key regulator of a series of genes involved in a number of cellular processes. In our experimental settings, we observed that lactate induces the most significant increase in mRNA levels of *CA9* compared to other evaluated HIF-1 target genes *VEGFA*, *SLC16A3* and *LDHA*. A similar pattern of slight effect of normoxic lactate on the expression profile of *VEGFA*, *PGK1* and *ANG* has been previously reported [41]. Authors showed that only 7 transcripts from 74 analyzed were upregulated more than 2-fold in response to lactate. The strongest effect of ascorbate dependent downregulation of HIF-1α target genes expression was also observed for *CA9* in head and neck carcinoma cell line 22B (more than 75-fold decrease in *CA9* compared to a 25-fold decrease for GLUT3 and a 50-fold decrease for MMP-2) [11]. Moreover, ascorbate almost completely inhibited the invasiveness of 22B cells through Matrigel [11]. These results indicate that *CA9* might represent one of the key effectors of lactate-induced HIF-1 activation. Indeed, dietary ascorbate supplementation of mice with B16-F10 xenografts decreased the level of HIF-1α protein in tumors and, in parallel, also the levels of CA IX, VEGF, and GLUT1 [47]. Increased ascorbate in tumors downregulated HIF-1 signaling and delayed tumor growth.

While HIF-1 is critical for *CA9* transcription [18], it is dispensable for the transcription of *VEGF* and merely serves as an enhancer of transcriptional activation under hypoxia [48]. Of important note, HIF-1 is not the sole transcription factor that is important to stimulate the expression of HIF-1 target genes, as their transcriptional regulation is determined by the combination of several transcription factors depending on the actual composition of the tumor microenvironment, including lactate availability. The microenvironment of normoxic high cell-density conditions activates signaling pathways, such as PI3K [10] and MAPK pathways [28], that phosphorylate HIF-1α, as well as other factors stimulating CA IX expression [49–51]. When appropriate microenvironmental conditions are met, lactate can efficiently enhance *CA9* transcription.

We further evidence that lactate influences CA IX at the protein level in both HeLa and SiHa cells, and that ascorbate reverses the effect of lactate-facilitated CA IX expression, which points to HIF-1 as the major mediator of lactate signaling. We observed higher increase in CA IX protein expression upon lactate treatment in SiHa compared to HeLa cells, especially after 24 h of treatment. Metabolic differences between HeLa and SiHa provide a possible explanation for the difference in the extent of lactate effect on CA IX. In HeLa cells, a high proportion of carbon derived from glucose (80%) is converted to lactic acid through glycolysis and only 4–5% enters the TCA cycle. Indeed, it has been reported that the major nutrient fuelling aerobic metabolism at the TCA cycle is glutamine, even in the presence of high glucose concentrations [52]. SiHa cells appear to be highly oxidative with limited lactate release, which indicates their possible use of glucose- and lactate-derived pyruvate for oxidative purpose [4, 32]. By stimulating oxidative metabolism and resulting ROS production (as described above), lactate can stabilize HIF-1α and influence CA IX levels more efficiently in SiHa compared to HeLa cells. Therefore, SiHa with their highly oxidative nature might be more responsive to the impact of lactate. Conversely, HeLa cells produce higher amounts of lactate than SiHa *via* glycolysis intracellularly and are thus potentially less responsive to extracellular lactate, especially when considering that MCTs are passive transporters driven by the gradient of lactate across cell membranes.

HRE alone is not sufficient for *CA9* induction by mild hypoxia in densely cultured normoxic cells. In high cell density-derived mild pericellular hypoxia, minimal levels of active HIF-1α and SP1 transcription factors are involved in the stimulation of *CA9* gene expression [10, 28]. Moreover, SP1 promoter binding has a prominent role in activating cell density-mediated CA IX expression [37]. Interestingly, our results show that HRE mutations caused only a minor decrease in *CA9* promoter activity compared to wt after 24 h of incubation at high density. This indicates that HIF-1 does not play a prominent role in the initial phases of normoxic CA IX induction. Instead, SP1 is the crucial factor. A 48 h normoxic incubation causes a gradual decrease in pericellular oxygen levels with increasing cellular density over time. This supports an increasing role of HIF-1 as evidenced by the more dramatic decrease in promoter activity of HREmut compared to wt.

We observed that extracellular lactate induces *CA9* expression only in normoxic conditions. Inability of either HIF-1 or SP1 to bind to the *CA9* promoter blocked the effect of lactate. We also observed that lactate stabilizes HIF-1α protein expression, but we did not observe a similar effect of lactate on SP1. Lactate did not increase SP1 levels after 24 h in SiHa; it even led to a decrease in SP1 expression after 48 h. The PR1 mutation that blocked the effect of lactate on *CA9* therefore reflects the importance of SP1 for normoxic *CA9* induction. A coordinative binding of HIF-1 and SP1 transcription factors is necessary for *CA9* expression in oxygenated cancer cells, and lactate augments the level of expression by increasing HIF-1α availability. Hypoxia massively stabilizes HIF-1α, and any additional effect of extracellular lactate could not be observed. Indeed, the metabolic switch induced by hypoxia would oppose lactate uptake *via* MCTs.

*CA9* and *VEGF* genes share similarities regarding transcriptional regulation and coordination between transcription factors. *CA9* and *VEGF* promoters do not contain a TATA box [33, 53]. Furthermore, clusters of SP1-binding sites are a typical feature of TATA-less promoters [54]. SP1 transcription factor is a key regulator of *VEGF* expression, and downregulation of SP1 binding to the *VEGF* promoter leads to strong inhibition of angiogenesis [33]. Another crucial factor regulating the transcription of *VEGF* in response to hypoxia is HIF-1 [33]. This underlines the relevance of a coordination of several transcription factors to drive efficient gene expression. Another example of HIF-1 and SP1 coordinated binding is found for the gene encoding lipid-sensitive transporter ABCA1, which comprises a HIF-1 binding site and SP1 conserved motifs within its promoter [55].

In this study, we further evidenced that the effect of lactate on CA IX is relevant not only *in vitro* but also *in vivo*. In SiHa xenograft cryosections, we observed the presence of MCT1 and CA IX in the same tumor areas. MCT1 is a critical component of lactate signaling in oxidative tumor cells, and its expression is important for lactate-induced angiogenesis and tumor growth *in vivo* [56]. Interestingly, Miranda-Gonçalves et al. showed that intracranial orthotopic tumors display strong colocalization of CA IX and MCT1, whereas most CA IX positive cells did not colocalize with MCT4, an outward lactate transporter preferentially expressed in hypoxic/glycolytic cancer cells [57, 58]. In an immunohistological study of basal-like breast cancer patients, MCT1, but not MCT4, was associated with GLUT1- and CA IX-positive tumor samples [59]. Clinical observations connect this highly glycolytic type of cancer with Myc-activation [60]. GLUT1 and MCT1, but not MCT4, are Myc-target genes [61, 62]. Additionally, advanced head and neck carcinomas display a significant correlation between hypoxically induced MCT4, GLUT1 and CA IX [63]. Despite an expected correlation of HIF-1α and CA IX expression, some CA IX-positive areas were found to be located far from pimonidazole-stained hypoxic areas where HIF-1α staining was almost absent. MCT1, which is expressed in both well-oxygenated and hypoxic regions of these tumors, showed an overall correlation and strong spatial relationship with CA IX. Thus, codistribution of CA IX with MCT1 in this heterogeneous environment could be a consequence of intermittent hypoxia, reflecting high CA IX stability and maintenance of its expression in re-oxygenated regions that can be further fueled by lactate imported by MCT1 [26, 64]. In 9 patients with non-small cell lung cancer, Hensley *et al.* demonstrated metabolic heterogeneity within tumors. They showed that the degree of tumor perfusion within the different areas of the tumor is related to the metabolic phenotype and respiratory fuel preference [65]. Glucose consumption was preferred in poorly perfused areas, whereas oxidation of nutrient sources other than glucose raised in well-perfused regions [66]. Their observations indicated that lactate is exploited by these tumors and that it supplies carbon to the TCA cycle. These data also suggest that tumor metabolism is crucially regulated by the local microenvironment and is dynamically changing in relationship to blood perfusion and the existence of intermittent hypoxia within tumors.

Genevestigator analysis confirmed that CA IX is co-expressed with MCT1 in areas with low MCT4 expression in several malignant neoplasms of different types. A largely represented group is brain tumors, more specifically oligodendromas and ependymomas. Oligodendromas account for only 4-5% of all primary brain tumors [67]. Mutations of isocitrate dehydrogenase (IDH)1 and IDH2 occur very frequently in grades II and III oligodendromas, but not in ependymomas [68]. These enzymes catalyze the conversion of isocitrate to α-ketoglutarate (α-KG) coupled with NAD(P)H production. α-KG is a cofactor for PDH. Thus, impairment of IDH reduces α-KG levels and promotes HIF-1α stabilization [69]. Furthermore, it decreases the production of NADPH, which may alter the redox state of the cells. IDH mutations might also be compensated by an increased glycolytic flux and lactate production [68]. Interestingly, neovascularization is a common feature of these neoplasms [70]. Analysis of 51 specimens of oligodendromas showed that 80.4% exhibited HIF-1α expression and that HIF-1α and microvessel density strongly correlated, which supports their significant vascularization that prevents hypoxia [71]. Thus, lactate generated by glycolysis can further support HIF-1α stabilization and CA IX expression in these types of vascularized tumors. Ependymoma is the third most common brain tumor type in children with a 50% probability of recurrence that results in death [72]. In an analysis of metabolic parameters in series of brain tumors, ependymomas demonstrated elevated glucose utilization compared to the overlying cortex and contralateral white matter [73, 74]. Glycolysis and lactate production can therefore represent a significant feature of ependymomas.

Several positive results of our Genevestigator search indicate that areas of high lactate uptake could be characterized by high CA IX expression. These areas are presumably oxygenated, as indicated by a low level of *SLC16A3*/MCT4 expression, which characterizes glycolytic cancer cells [9]. Additional studies also proved this expression pattern. Small cell lung cancer biopsy specimens from 78 patients showed that 21% of cases exhibited high CA IX and MCT1 expression, but low MCT4 expression [75]. Strong overall and spatial correlation of MCT1 and CA IX was further observed in head and neck and breast carcinoma samples [59, 63].

In conclusion, tumors are heterogeneous entities in many aspects, including oxygenation. Lactate is a by-product of glycolytic metabolism triggered by hypoxia. However, it is not a dead-end metabolic product, but rather supports a metabolic symbiosis between hypoxic and oxygenated tumor cells [4, 7]. By stabilizing HIF-1α, lactate serves as a hypoxia mimetic in oxygenated cells or cells exposed to fluctuating hypoxia. In this study, we provide evidence that CA IX, one of the major constituents of aggressive behavior of tumor cells, is induced by extracellular lactate in oxygenated cancer cell lines *in vitro* and *in vivo*. Lactate can therefore sustain the aggressive behavior of cancer cells in distant sites by various mechanisms, possibly including stimulation of CA IX expression.

## MATERIALS AND METHODS

### Cell lines, treatment and reagents

HeLa human cervix cancer adenocarcinoma cells and SiHa human cervix squamous cell carcinoma cells were from ATCC. Cells were routinely cultured in DMEM containing 4.5 g/l glucose, 10% fetal calf serum (Lonza BioWhittaker) and gentamicin (Sandoz) at 37°C in humidified air with 5% CO_2_. Prior to treatment, cells were kept in sparse culture conditions for one week in order to reduce cell-density-induced CA IX expression. After a week, cells were plated at high density (100,000 cells/cm^2^) in order to stimulate CA IX expression in normoxia. Attached cells were incubated for 24 h or 48 h in basal DMEM (Sigma Aldrich) supplemented with 1g/L of glucose (Sigma Aldrich), 2 mM UltraGlutamine (Lonza), 1% fetal calf serum, 10 mM NaHCO_3_ (Sigma Aldrich), gentamicin, phenol red and no pyruvate. Cells were incubated without (control) or with 10 mM sodium *L*-lactate (Sigma Aldrich) or 10 mM sodium *L*-lactate together with 100 μM sodium *L*-ascorbate (Sigma Aldrich).

### Immunoblotting

Cells were rinsed with PBS and lysed in RIPA buffer (50 mM Tris/HCl pH 7.4, 150 mM NaCl, 1% Triton X-100 in PBS, 0.05% sodium deoxycholate in PBS, 1 mM EDTA, 0.1% sodium dodecyl sulfate) containing a cocktail of protease inhibitors (Roche). Proteins were quantified by the BCA kit (Pierce), separated on 8% SDS-PAGE gels under reducing conditions and transferred onto a PVDF membrane. Target proteins were detected with the following primary antibodies: monoclonal mouse anti-HIF-1α (BD Biosciences), polyclonal goat anti-β-actin (Santa Cruz Biotechnology), monoclonal mouse anti-CA IX M75 diluted in hybridoma medium [76], polyclonal rabbit anti-SP1 (Millipore), and monoclonal mouse anti-Hsp90 (BD Biosciences). Secondary antibodies were: polyclonal goat anti-mouse peroxidase-conjugated IgG (Sigma Aldrich), polyclonal rabbit anti-goat peroxidase-conjugated IgG (Dako), and polyclonal goat anti-rabbit peroxidase-conjugated IgG (Sigma Aldrich). Signal was quantified using the ImageJ software (NIH).

### Lactate measurement

Media from control normoxic and hypoxic cells were collected after 24 h for measurement of extracellular lactate. Media were filtered and extracellular lactate was measured enzymatically using CMA 600 analyzer (Aurora Borealis). Media incubated without cells were used to measure initial concentration of lactate. Extracellular lactate was determined by the difference between media with cells and media that contained no cells.

### RNA extraction, reverse transcription and RT-qPCR

Total RNA was extracted using Instapure reagent (Eurogentec) and transcribed to cDNA by the High Capacity cDNA Reverse Transcription kit (Applied Biosystems). Quantitative PCR was performed with PowerSYBR Green PCR Master Mix on a StepOne Real-Time PCR system (Applied Biosystems) with initial denaturation at 95°C for 10 min followed by 40 cycles of denaturation at 95°C for 15 sec and annealing at 60°C for 1 min. All reactions were performed in triplicates and repeated three times. qPCR was performed with the following primers:

**Table:** 

*ACTB*/β-actin	sense, 5′-TCCTCCCTGGAG AAGAGCTA-3′
	antisense, 5′-ACATCTGCTGG AAGGTGGAC-3′
*CA9*	sense, 5′-AGTGCCTATGA GCAGTTGCT-3′
	antisense, 5′-TAGCCGAG AGTCACCAGGTC-3′
*SLC16A3/*MCT4	sense, 5′-CTCACCATCCTGG GCTTCAT-3′
	antisense, 5′-AGAAGAAGT TGCCCAGCAGCA-3′
*LDHA*	sense, 5′-TGGCAGCCTTT TCCT TAGAA-3′
	antisense, 5′-ACTTGCAGTT CGGGCTGTAT-3′
*VEGFA*	sense, 5′-CTTGCTGCTC TACCTCCACCAT-3′
	antisense, 5′-CACACAGG ATGGCTTGAAGATG-3′

### Transfection and dual luciferase assay

All human promoter constructs were generated by insertion of PCR-amplified -174/+37 *CA9* genomic fragments upstream of the firefly luciferase gene in pGL3-Basic luciferase reporter vector (Promega, Madison, WI, USA) [28]. pRL-TK *Renilla* vector (Promega) served as a transfection efficiency control. *CA9* promoter constructs with mutations in HIF-1 binding site (HREmut), SP1 binding site (PR1mut) and AP1 binding site (PR2mut) were cloned into pGL3-Basic luciferase reporter vector from the original expression plasmid prepared and described previously [37, 38]. Constructs contained the following mutations (bold, underlined):

HREmut, mutations in HIF-1-binding site: TTTCCAATGC**TTT**TACAGCCCG;

PR1mut, mutations in SP1 binding site: AGGCTTGCTCCT**AA**CCCACCAG;

PR2mut, mutations in AP1 binding site: CGCTCTGTGAGT**TG**GCCTGCTCCC.

Cells were plated into 35-mm-diameter Petri dishes to reach 50-90% density on the following day. Transient transfection was performed with 2 μg of promoter-containing luciferase construct and 100 ng of pRL-TK plasmid DNA using *Trans*IT-2020 reagent (Mirus) according to the manufacturer's recommendations. The day after transfection, cells were trypsinized and plated in quadruplicates into 24-well plates. Cells were allowed to attach until the following day, and were then treated with 10 mM of sodium *L*-lactate and either transferred to 2% hypoxia or maintained in normoxia for an additional 24 h and 48 h. Reporter gene expression was assessed using Dual-Luciferase Reporter Assay System (Promega), and luciferase activity was normalized against *Renilla* activity.

### *In vivo* experiments

For CA IX expression analyses in SiHa xenografts, we used samples collected in a previous study [40]. These *in vivo* experiments were performed with the approval of the *Université catholique de Louvain* (UCL) authorities (specific approval ID was TUMETABO) according to national animal care regulations. Immunoblotting was performed as described above.

### Immunofluorescent staining of xenografts cryosections and confocal microscopy

Lactate-treated SiHa xenograft cryosections [40] were also used for immunofluorescent analysis. Cryosections (5-μm) were fixed with ice-cold acetone and air-dried. Prior to staining, sections were rehydrated in PBS 3 × 3 min. Blocking of nonspecific antibody binding sites was performed in 10% horse serum in PBS containing 0.05% Tween 20 (PBST) for 15 min at room temperature (RT). Sections were briefly washed twice in PBST supplemented with 0.1% BSA. Primary antibodies were polyclonal goat anti-human CA IX (R&D Systems) and polyclonal rabbit anti-MCT1 (Millipore). Sections were incubated with primary antibodies diluted in 0.5% BSA in PBST at 4°C overnight. After washing 3 × 5 min in 0.02% Tween 20 in PBS, fluorescent secondary antibodies (Alexa Fluor 594 rabbit anti-goat IgG and 555 goat anti-rabbit IgG; Thermofisher) were diluted in 0.5% BSA in PBST and applied onto the sections in the dark for 1h at RT. Incubation with secondary antibodies omitting primary antibodies was used as negative control. Following washing 1 × 10 min in 0.02% Tween 20 in PBS and 3 × 5 min in PBS, cryosections were mounted onto slides with the Duolink In Situ Mounting Medium containing DAPI (Sigma Aldrich). Images were acquired on a LSM510 Meta confocal microscope in multitrack mode at 200x magnification.

### In silico co-expression analysis

Gene expression data from various datasets were obtained from Genevestigator (https://www.genevestigator.com/gv/). Cancer-specific genes *CA9*, *SLC16A1*/MCT1 and *SLC16A3/*MCT4 were analyzed with Genevestigator's condition search tool “Cancers”. We selected neoplasms with relatively high *CA9* and *SLC16A1* expression compared to SLC16A3 expression. Results are given as heatmaps in different color codes that reflects % of expression potential. The color scale with heatmaps is given in log2 ratio values.

### Statistical analyses

Data are presented as means ± SEM of three independent experiments. Student's *t* test and one-way ANOVA (Tukey's *post hoc* test) were used where appropriate. p < 0.05 was considered to be statistically significant.
